# Accumulation of HIV-1 drug resistance in patients on a standard thymidine analogue-based first line antiretroviral therapy after virological failure: implications for the activity of next-line regimens from a longitudinal study in Mozambique

**DOI:** 10.1186/s12879-017-2709-x

**Published:** 2017-09-05

**Authors:** Andrea De Luca, Zita Jorge Sidumo, Giacomo Zanelli, Noorjehan Abdul Magid, Richard Luhanga, Davide Brambilla, Giuseppe Liotta, Sandro Mancinelli, Maria Cristina Marazzi, Leonardo Palombi, Susanna Ceffa

**Affiliations:** 1 UOC Malattie Infettive Universitarie, AOU Senese and Department of Medical Biotechnologies, Siena University Hospital, Viale Bracci 16, 53100 Siena, Italy; 2 DREAM Program, Maputo, Mozambique; 3 DREAM Program, Malawi and UPO (Università del Piemonte Orientale), Novara, Italy; 4 DREAM Program, Rome, Italy; 5 University of Tor Vergata, Rome, Italy; 6 University LUMSA, Rome, Italy

**Keywords:** HIV-1, Antiretroviral drug resistance, Second-line ART, Virological failure, HIV-1 RNA

## Abstract

**Background:**

We describe the accumulation of HIV-1 drug resistance and its effect on the activity of next-line components in patients with virological failure (HIV-1 RNA >1000 copies/mL) after 1 year (t1) of first-line antiretroviral therapy (ART) not switching to second-line drugs for one additional year (t2) in low-middle income countries (LMIC).

**Methods and results:**

We selected 48 patients from the DREAM cohort (Maputo, Mozambique); their median pre-ART CD4+ cell count was 165 cells/μl. At t1 patients were receiving ART since a median of 12.2 months (mainly zidovudine/lamivudine/nevirapine), their median HIV RNA was 3.8 log10 copies/mL, 43 (89.6%) presented at least one resistance-associated mutation (RAM), most frequently for lamivudine/emtricitabine, nevirapine and efavirenz. Resistance to tenofovir, was 10% at 1 year and higher than 20% at 2 years, while projection at 3 years was >30%. At t2, 42 (89.4%) had a predicted low-level or higher resistance to at least 1 s-line drug. At t1, the frequency of RAM in patients with a lower adherence to pharmacy appointments (<95%) was significantly lower (12/20, 60% for NRTI and 14/20, 70% for NNRTI) than in those with a better adherence (26/28, 92.8% for NRTI and 25/28, 89.3% for NNRTI) (OR 0.12, 95% CI 0.02–0.63, *p* = 0.012 and OR 0.28, 95% CI 0.06–1.29, *p* = 0.103, respectively). Overall thymidine analogue mutations (TAMs) accumulation rate was 0.32/year, 0.50/year in the subgroup with HIV RNA >10,000 copies/mL; NNRTI RAM accumulation rate was 0.15/year, 0.40/year in the subgroup with HIV RNA >10,000 copies/mL.

**Conclusions:**

While the activity of NNRTIs is compromised early during failure, tenofovir and zidovudine activity are reduced more frequently after 1 year of documented virological failure of thymidine analogue-based first-line ART, with RAMs accumulating faster in patients with higher viral loads. The present observation may help informing decisions on when to switch to a second line ART in patients on virological failure in LMIC.

## Background

As of December 2016, 19,5 million people were receiving antiretroviral therapy (ART) word-wide. As antiretroviral treatment is rapidly being scaled up, HIV drug resistance is emerging and increasing at the global level. WHO recently reported that levels of pre-treatment drug resistance (PDR) to nevirapine and efavirenz, the two most affordable non-nucleoside reverse transcriptase inhibitors (NNRTIs) used in the first-line ART in resource-limited settings, was 10% or above in 6 of 11 countries performing nationally representative HIV drug resistance (DR) surveys, 3 out of 4 in Africa [1]. According to a systematic review, the estimated annual incremental rate of PDR to NNRTIs ranged between 15% and 29% in Africa [2]. In addition, NNRTI resistance among people with unsuppressed viral load on first-line NNRTI regimens ranged from 47% to 90%. A systematic review of studies of acquired drug resistance (ADR) in adults from 30 low-middle income countries published between 2014 and 2016 found that 68% of those failing NNRTI regimens had one or more resistance mutation after a median time on ART of 15 months. Resistance was more frequent for NNRTI (61%), as for nucleoside/nucleotide reverse transcriptase inhibitors (NRTI) (55%) [1]. Viral load monitoring is recommended by WHO in order to diagnose and confirm ART failure and provide an early and more accurate indication to switch treatment from first-line to second-line drugs, reducing the accumulation of resistance mutations [3]. Lower viral load is associated with less HIV-1 resistance mutations to all drug classes and viral load monitoring is expected to significantly reduce the emergence of acquired HIV drug resistance [3–8]. WHO consolidated HIV guidelines recommend a NNRTIs-based regimen as first line ART [3]. After failure patients may be switched to boosted protease-inhibitors (PIs)-based second-line ART [3]. However, given the paucity of next treatment lines in low-middle income countries (LMIC), the correct switching time should, among others, be informed by the probability of accumulating resistance to the NRTI agents included in the second line at a given time of virological failure [3, 6–9]. Objectives of this study are to describe the accumulation of HIV-1 drug resistance and its effect on the activity of next-line therapy components in patients undergoing viral load monitoring and showing virological failure after 1 year of first-line ART but not switching to second-line ART for one additional year.

## Methods

A retrospective longitudinal cohort study was performed with parameters extracted from the Drug Resource Enhancement against AIDS and Malnutrition (DREAM) database for HIV-infected patients. DREAM is a program of care for people with HIV designed and managed by the Community of Sant’Egidio (Italy), which is now working in ten African countries including Mozambique [3, 10]. The DREAM program offers free of charge state-of-the-art treatment, diagnostic facilities and nutritional supplementation to all HIV- infected patients within centers from the public sector [3, 10]. Antiretroviral therapy is supplemented by the governmental program following National guidelines. Patients on ART perform plasma viral load monitoring at yearly intervals. We selected patients from 3 sites in Maputo (Mozambique) who a) were on a first-line ART b) had an HIV RNA >1000 copies/mL after 1 year (**t1**) and after 2 years (**t2**) of ART without switching to a second-line regimen and c) had a stored plasma sample at t1 and t2. We also considered: **t2^**: 2 years after ART initiation (1 year after first documented virological failure) considering historical genotype; t3*: 3 years after ART initiation (2 years after first documented virological failure), projected results assuming a continuous linear accumulation of resistance mutations and historical genotype at year 2 (cumulating resistance of t1 + t2). Genotyping of reverse transcriptase (RT) and protease was performed at t1 and t2 by the Trugene HIV-1 genotyping method (Siemens Health Care Diagnostics, NY, USA). Proportion of subjects with major resistance associated mutations (RAM) (International Antiviral Society-USA list 2014) to NRTIs, thymidine analogue mutations (TAMs) and NNRTI RAM and with resistance to individual drugs as interpreted by Stanford’s HIVdb 7.0 system (considering at least low level resistance, LLR to each drug) was calculated. Adherence was measured by per cent on time pharmacy appointment keeping, allowing a delay tolerability of 10 days. The protocol was approved by the National Ethics Committee from Mozambique (approval n. 384/CNBS/12 on October 18, 2012).

### Statistical analysis

Baseline characteristics of study patients were described using standard descriptive statistics. Associations between socio-demographic and HIV-related variables were assessed using the Chi-square test for categorical variables and Student’s t-test or the Mann – Whitney test for continuous variables, as required. Association between adherence to pharmacy appointments and resistance mutations was analyzed by logistic regression. Data were analyzed using SPSS software package (version 18.0 Chicago, IL).

## Results

**Fig. 1:**
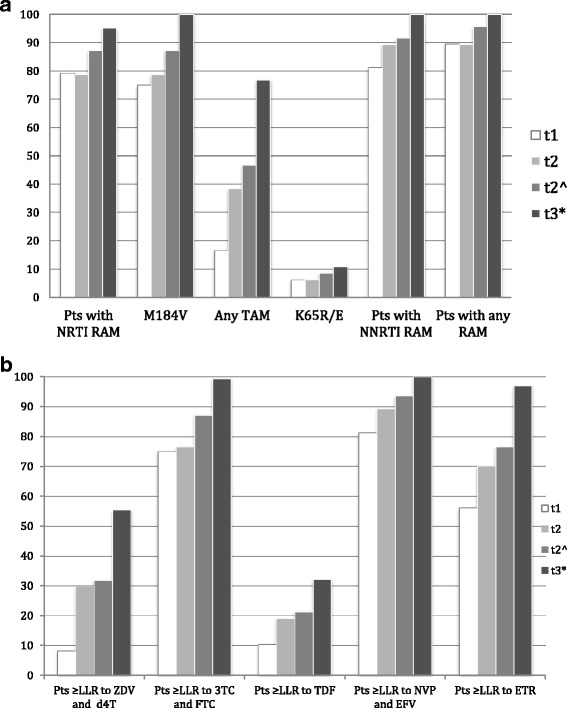
**a** Proportion of patients with major resistance mutations at t1* (first documented HIV RNA >1000 copies/mL at 1 year of ART) and t2 *(continued HIV RNA >1000 copies/mL at 2 years of ART). t2^ represents the cumulative resistance summing resistance at t1 and t2. t3 represents the projection of resistance at 3 years based on t1 and t2^ and assuming absence of resistance at baseline. **b** Proportion of patients with predicted resistance to individual drugs at t1 and t2 and the projection at 3 years. * t1 total patients *n* = 48, t2 total patients *n* = 47; NRTI, nucleoside/nucleotide reverse-transcriptase inhibitor; NNRTI, non-nucleoside reverse-transcriptase inhibitor; PIs, protease inhibitors; RAM, resistance-associated mutations; TAM thymidine analog mutation; LLR, low-level resistance according to the interpretation by hivdb v 7.0; ZDV, zidovudine; 3TC, lamivudine; TDF, tenofovir; NVP, nevirapine; EFV, efavirenz; ETR, etravirine

There were 48 eligible patients enrolled in the study: 24 (50%) were males. At baseline (time of ART initiation, median calendar year 2010) the median patients’s age was 35 years (inter-quartile range, IQR 28.5–37.7), the median CD4+ cell count was 165 cells/μl, and the mean viral load was 4.69 log_10_ copies/ml. Most of the patients (95.8%) were infected with HIV-1 subtype C. At t1, patients were receiving ART for a median of 12.2 months and the most frequently employed regimen was zidovudine/lamivudine/nevirapine (*n* = 36, 75%), followed by stavudine/lamivudine/nevirapine (*n* = 4, 8.3%), stavudine/lamivudine/efavirenz (*n* = 3, 6.2%), zidovudine/lamivudine/abacavir (*n* = 3, 6.2%) and zidovudine/lamivudine/efavirenz (*n* = 2, 4.2%). Between t1 and t2, NRTI components were substituted in 2 cases from zidovudine/lamivudine to stavudine/lamivudine, in 2 cases from stavudine/lamivudine to zidovudine/lamivudine and in 2 cases from stavudine/lamivudine to tenofovir/lamivudine. Table 1 shows the main clinical and virological characteristics of the patients at time t1 and t2. Out of 48 patients, 43 (89.6%) presented at least one RAM after one year of ART and 34/48 (70.8%), showed at least one RAM both for NRTI and NNRTI. The proportion of RAM showed an increase from t1 to t2 (Fig. 1a). In relation to individual drugs the most frequent resistance mutations were observed for lamivudine and emtricitabine, nevirapine and efavirenz with progressive accumulation over time (Fig. 1b). Resistance to tenofovir, which is the cornerstone drug of the NRTI backbone therapy for the second-line regimen in patients failing a first-line regimen containing thymidine-analogues was 10% at 1 year and slightly higher than 20% at 2 years, when cumulative resistance was considered; projection at 3 years showed a > 30% probability of tenofovir resistance. Two of 47 (4.3%) patients with a protease sequence available had major PI resistance mutations (I54T and L90 M each in one patient), conferring LLR to atazanavir/r and lopinavir/r. We then considered the predicted resistance to the candidate second-line regimens based on WHO recommendations and the type of first-line regimen failing. At t1 31 (64.6%) had at least LLR to 1 second-line drug and another 5 (10.4%) to 2 drugs, so that a total of 36 (75%) had at least 1 second-line drug with predicted low-level or higher resistance. At t2 31 (66.0%) had at least LLR to 1 second-line drug and another 11 (23.4%) to 2 drugs, so that a total of 42 (89.4%) had at least 1 second-line drug with predicted low-level or higher resistance.

**Table 1:** Clinical and virological follow-up at t1 (*n* = 48 patients) and t2 (*n* = 47 patients)

	t1	t2
CD4 count (cells/μl)^a^	234 (113–322)	236 (136–386)
HIV RNA (log_10_ copies/ml)^a^	3.8 (3.5–4)	3.9 (3,5–4.5)
Hb (g/dL)^a^	12.6 (11.3–13.1)	12.1 (11.2–13.3)
BMI (Kg/m^2^)^a^	22.2 (20.7–25.3)	21.9 (20.5–24.7)
Adherence (%)^a^	100 (92–100)	100 (92–100)
Percent of patients with <95% adherence to pharmacy appointments	41.7	41.3

*BMI* Body mass index

^a^median (IQR)

At t1, the probability of RAM in patients on virological failure differed based on levels of adherence to pharmacy appointments. In particular, frequency of NRTI RAM in patients with a lower adherence to pharmacy appointments (<95%) was significantly lower (12/20, 60%) than in those with a better adherence (26/28, 92.8%) (OR 0.12, 95% CI 0.02–0.63, *p* = 0.012). The mean yearly TAMs accumulation rate was 0.32; which rose to 0.49 in patients with pharmacy appointment adherence >90%. In patients with HIV-1 RNA > 10,000 copies/mL TAMs showed a mean yearly accumulation rate of 0.50. Probability of NNRTI resistance was also lower in less adherent patients at t1 (14/20, 70% vs 25/28, 89.28%; OR 0.28, 95% CI 0.06–1.29, *p* = 0.103). Mean NNRTI RAMs accumulation rate was 0.15/year. In subjects with pharmacy appointment adherence >90% the mean accumulation rate was 0.17/year. In patients with HIV-1 RNA > 10,000 copies/mL the mean yearly accumulation rate of NNRTI RAMs was 0.40.

## Discussion

Mozambique, a sub-Saharan country with HIV prevalence of 10.6% provides antiretroviral therapy based on a public health approach [3, 11, 12]. Treatment options for HIV infected people in low-middle income countries (LMIC) are based on WHO guidelines [3]. First-line regimen includes two NRTI (tenofovir or zidovudine and lamivudine or emtricitabine) plus one NNRTI (nevirapine or efavirenz) whereas a boosted protease inhibitors-based ART is used as second line regimen with a substitution of the NRTIs: tenofovir is given after zidovudine failure, while zidovudine is recommended in the second-line after tenofovir has failed in the first-line regimen [3].This treatment sequencing strategy is based on the rationale that after virological failure with 2 NRTI + a NNRTI, the activity of the protease inhibitors is preserved and the cross-resistance of the alternate NRTIs is limited [3, 6, 13]. However, after tenofovir failure, HIV-1 usually selects for RAMs such as K65R that do not affect zidovudine activity; on the contrary zidovudine selects for TAMs that accumulate and progressively confer increasing cross-resistance to tenofovir. This is one of the reasons why WHO now recommends to prefer tenofovir as first-line regimen, but many countries still use zidovudine due to cost and procurement issues. Viral load monitoring to detect treatment failures, is now recommended by WHO and, although not yet available in many areas, is becoming increasingly accessible [3]. Consideration of the timing of treatment switch after 1st-line ART failure is particularly important in resource-limited settings where salvage regimens are scarce and costly [9]. For these reasons in LMIC, the correct switching time should also be informed by the probability of accumulating resistance to the subsequent treatment lines at a given time of virological failure [7, 9, 13]. In this study, we report how RAM accumulate after virological failure of a thymidine analogue-based first-line regimen in a resource-limited setting. In particular, we show that cross-resistance to tenofovir was still limited when failure was detected at 1 year after ART initiation. After 2 years and, in projection, after 3 years, cross-resistance to tenofovir accumulated significantly. This lead to a significant accumulation of resistance to drugs that, based on WHO guidelines, would have been used for the second-line regimen: a predicted low-level or higher resistance to at least 1 drug of the second-line regimen rose from 75% of cases at t1 to 89% at t2, while 10% at t1 and 23% at t2 showed a predicted resistance to 2 second-line drugs. Previous studies have anayzed the accumulation of drug resistance mutations in patients failing first-line regimens in Sub-Saharan Africa. In a retrospective study in South Africa, in 43 patients performing sequential resistance tests with a median interval of 5 months, RAMs accumulated at a mean of 0.07/month of drug exposure [14]. In a prospective cohort of Zambian children on first-line ART, 6 had sequential genotypes while failing on stavudine/lamivudine/nevirapine and showed an accumulation rate of 0.59 TAMs/year [15]. In a retrospective analysis of a randomized study performed in African countries on 36 genotype pairs from weeks 48 and 96 of first-line ART, the mean TAMs accumulation rate was 1.50/year in nevirapine-treated participants and 1.82/year in abacavir-treated participants [16]. In a retrospective analysis of adults and children failing NNRTI-based first-line ART, NNRTI resistance mutations accumulated at 0.62/year and NRTI resistance mutations at 0.84/year [17]. In our study we observed a mean TAM accumulation rate of 0.32/year; which rose to 0.49/year in patients with a pharmacy refill adherence >90%. The yearly accumulation rate of NNRTI resistance mutations was 0.15, rising at 0.17 in adherent patients. An hypothesis for the reason for the lower rate of resistance accumulation in this cohort as compared to previous reports may be the availability of viral load monitoring. Indeed, patients included here were selected among those not switching to second-line for one year despite documented virologic failure. The population included showed relatively low viral loads at failure, and this might have been a reason for keeping them on first-line, prompting adherence interventions before switching to second-line. This probably selected a population at lower risk of resistance accumulation, as reported by other studies relating drug resistance accumulation to viral load [4, 18]. In agreement with this, patients with an HIV-1 RNA >10,000 copies/mL in this study showed higher yearly accumulation rates for TAMs and NNRTI resistance mutations (0.50 and 0.40, respectively), values that are closer to those provided by previous reports. Our findings may represent a practical indication for the management of patients with virological failure in this settings. In particular, after initial detection of virological failure patients may still benefit from adherence counselling strategies without major risk of accumulating significant cross-resistance to second-line drugs. However, the risk of resistance accumulation is higher in patients with an HIV-1 RNA above 10,000 copies/ml, and if virological failure persists subsequently, despite adherence implementation, a switch to second-line ART should be recommended, as RAM will accumulate in the majority of patients. Our findings should be interpreted with caution given the limited sample size and the retrospective design of the study and require validation in a prospective study. Moreover, our sequencing technique did not cover the mutation N348I in the connection domain, which is frequently selected by nevirapine and thymidine analogues in subtype C, and slightly reduces susceptibility to NNRTIs and zidovudine, so our analyses may have slightly underestimated resistance to these agents [19]. However, the accurate patients selection and follow up and the contemporary longitudinal assessment of viral load, resistance and adherence at specified times on ART represent strong points of this cohort study. It is important to consider that studies in Sub-Saharan Africa have shown that viral load could be successfully re-suppressed with boosted PI-based second-line regimens despite the presence of NRTI resistance after first-line failure. One observational cohort study showed successful virologic suppression with second-line drugs, despite 53% were predicted to receive partially active regimens due to drug resistance [20]; moreoever, in a randomized clinical trial [21] response to a second-line regimen based on boosted PI +2–3 NRTIs was better as compared to a boosted PI + raltegravir or boosted PI monotherapy despite NRTIs had no predicted activity due to resistance mutations; finally, in another randomized trial, the major determinant of response to boosted PI +2–3 NRTIs as second-line regimen was adherence but not baseline resistance [22]. Therefore, our findings on NRTI RAM accumulation on first-line failing patients may have limited implications for clinical practice given the residual activity of a regimen based on boosted PI + NRTIs. It remains to be established whether the introduction of dolutegravir in the recommended first-line or second-line regimens will change this scenario.

## Conclusions

While the activity of NNRTIs is compromised early during failure, tenofovir and zidovudine activity begin to be reduced more frequently after 1 year of documented virological failure of thymidine analogue-based first-line ART. Higher viral load at failure is associated with a faster rate of resistance accumulation. The present observation may inform decisions on when to switch to a second line therapy in patients on virological failure in LMIC settings.

## Abbreviations

3TC: Lamivudine
ADR: Acquired drug resistance
ART: Antiretroviral therapy
DREAM: Drug Resource Enhancement against AIDS and Malnutrition
EFV: Efavirenz
ETR: Etravirine
HIV: Human immunodeficiency virus
LLR: Low-level resistance
LMIC: Low-medium income countries
NNRTI: Non-nucleoside reverse-transcriptase inhibitor
NRTI: Nucleoside/nucleotide reverse-transcriptase inhibitor
NVP: Nevirapine
PDR: Pre-treatment drug resistance
PIs: Protease inhibitors
RAM: Resistance-associated mutations
TAM: Thymidine analog mutation
TDF: Tenofovir
WHO: World Health Organization
ZDV: Zidovudine
